# Metabolomics of aging assessed in individual parasitoid wasps

**DOI:** 10.1038/srep34848

**Published:** 2016-10-07

**Authors:** Apostolos Kapranas, Charles J. P. Snart, Huw Williams, Ian C. W. Hardy, David A. Barrett

**Affiliations:** 1 School of Biosciences, University of Nottingham, LE12 5RD, UK; 2 Centre for Analytical Bioscience, School of Pharmacy, University of Nottingham, NG7 2RD, UK; 3 School of Chemistry, University of Nottingham, NG7 2RD, UK

## Abstract

Metabolomics studies of low-biomass organisms, such as small insects, have previously relied on the pooling of biological samples to overcome detection limits, particularly using NMR. We show that the differentiation of metabolite profiles of individual 1 mg parasitoid wasps of different ages is possible when using a modified sample preparation and a combination of untargeted NMR and LC-MS based metabolomics. Changes were observed between newly emerged and older wasps in glycerolipids, amino acids and circulatory sugars. This advance in chemical profiling has important implications for the study of the behaviour and ecology of parasitoids and many other species of small organisms because predictions and observations are typically made at the level of the individual. Thus, the metabolomic state of low-biomass individuals can now be related to their behaviour and ecological performance. We discuss specifically the utility of age-related metabolomic profiling but our new approach can be applied to a wide range of biological research.

The study of insect and arthropod systems has played a considerable role in the development of modern behavioural and evolutionary ecology, chiefly through the use of empirical studies to observe behaviour at the level of individual organisms^1^. This is due to the marked influence that the physiological state of an individual can have on their behaviour as it varies under a range of biotic and abiotic factors^2^. These factors similarly influence the metabolome of an individual organism, the alteration of which has been demonstrated to both directly and indirectly affect organism fitness and behaviour^3^,^4^.

The use of metabolomic analysis of insects to complement ecological/behavioural studies has increased rapidly over the last decade, coinciding with advances in both nuclear magnetic resonance (NMR) and mass spectrometry (MS) based methodologies^5^. The ability to analyse individual insects has, however, been limited by the difficulty in detecting metabolites in low biomass insects, such as the fruit fly *Drosophila melanogaster* which is an important model organism. In the few cases that the analytical sample equated to a single intact organism, and solely using LC-MS, the study either targeted a limited range of metabolites, such as branched amino acids in *D. melanogaster*^6^, or concerned broadly water-soluble metabolites in larger insects such as flesh flies and stoneflies^7^,^8^. Where target insects were smaller in size, pooling of multiple low biomass organisms to obtain sufficient sensitivity for analysis has often been necessary^9^,^10^,^11^,^12^,^13^,^14^,^15^. Whole organism sample pooling imposes two major experimental limitations: the loss of statistical power associated with the reduction of the number of replicates within an experiment^16^, and the loss of information regarding how inter-individual variation in metabolite concentrations may affect an organism’s behavioural state. While the first limitation could be overcome by using more individual organisms in pooled groups, this becomes impractical where tens or hundreds of organisms are required, for example in behavioural experiments, and this does not address the second limitation. It is therefore desirable to generate metabolomics data from individual organisms, rather than pooled samples, when behavioural/developmental data are acquired from experimental studies involving individual insects. There has also been tremendous interest in developing new methods for determining the age of individual insects, particularly for species of medical importance, such as mosquitoes^17^. Considerable effort has gone into molecular approaches, particularly transcriptomics and proteomics^18^,^19^,^20^,^21^. Metabolomics approaches have been used less frequently, but have been successfully used for developing biomarkers of aging in a variety of model organisms, such as *C. elegans*, mice and *Drosophila*^22^.

The low individual biomass of many insects commonly used in behavioural studies (e.g. *D. melanogaster*^23^, mosquitoes^24^) poses a significant methodological challenge particularly for NMR and also with MS-based metabolomic analysis. By combining modified sample extraction and concentration methodologies with the use of high-field/high sensitivity instrumentation, we believe it is possible to derive reliable and high quality analytical NMR and MS data from individual insects of very low biomass and thus contribute significantly to the physiological/biochemical understanding of insect behaviour. The use of NMR in particular relies on high sample concentration to compensate for a lack of sensitivity, especially when compared to MS^25^. Currently no metabolomic studies exist that have been carried out on single low-biomass insects, most likely due to the difficulty in generating highly concentrated, high volume samples from low-biomass organisms. Existing pooled low-biomass insect metabolomic approaches have largely been restricted to the use of either gas chromatography (GC) or liquid chromatography (LC) MS^5^, though two NMR studies do exist^26^,^27^. Low-biomass insect analytical studies based on pooling biological samples have previously examined the arctic midge, *Belgica antarctica*^7^,^28^, *Anopheles* mosquitoes^29^, drosophilid flies^13^,^14^,^15^, aphids^26^,^27^ and the parasitoid wasps *Praon volucre*^30^ and *Venturia canescens*^31^. Previous non-insect studies have attempted to reconcile NMR techniques with analysis of low-biomass organisms^32^,^33^. Nagato *et al*.^33^ examined the potential of a microprobe technique for analysis of single *Daphnia magna*, a cladoceran freshwater flea with a whole organism mass of approximately 0.2 mg. While multivariate comparisons of individual sample extraction methodologies were performed, Nagato *et al*.^33^ did not seek to further validate the approach through the analysis of metabolic variations between two or more differential treatment states. Similarly, a study on minute roundworms, *Caenorhabditis elegans*, did not provide any further validation of the NMR generated metabolome of a single worm^32^.

As part of our ongoing behavioural studies on the parasitoid wasp, *Goniozus legneri* Gordh (Hymenoptera: Bethylidae) (Supplementary Figure S1), we required complementary whole-organism metabolomic data from individual insects. These wasps have been utilised to examine the mechanisms underlying clutch size decisions, developmental mortality, sex allocation decisions, and dyadic contests^34^,^35^. An individual female *G. legneri* wasp has a total biomass in the range of 0.5–2 mg. Previous work has also confirmed that adult *Goniozus* wasps are incapable of *de novo* lipogenesis^36^. As it has been confirmed that the total lipid reserves of *Goniozus* wasps will decline over time after adult eclosion, this provides an excellent opportunity to evaluate potential approaches to the analysis of low-biomass samples. Alongside decreases in large storage lipids, starvation related declines in polar metabolites, including amino acids and circulatory sugars, should be observable by a combined NMR and LC-MS approach. As such, the metabolomic analysis of the individual wasps was required to cover as broad a range of physiochemical properties of metabolites as possible, ranging from highly polar and water soluble molecules (such as sugars and amino acids) to water insoluble compounds (such as lipids).

Here we demonstrate a new application for generating NMR and MS metabolomic datasets based on single insect analysis, which is made possible by optimising existing sample extraction procedures. Unlike previous low-biomass metabolomics studies, this approach aims simultaneously to measure both polar and lipid metabolites by employing a modified Bligh and Dyer^37^ extraction coupled with NMR and LC-MS analysis. We use these established methods to generate metabolite profiles of individual *G. legneri* and to reveal the metabolic effects of aging in this organism.

## Results

### Metabolite profiling using NMR

**Figure 1:**
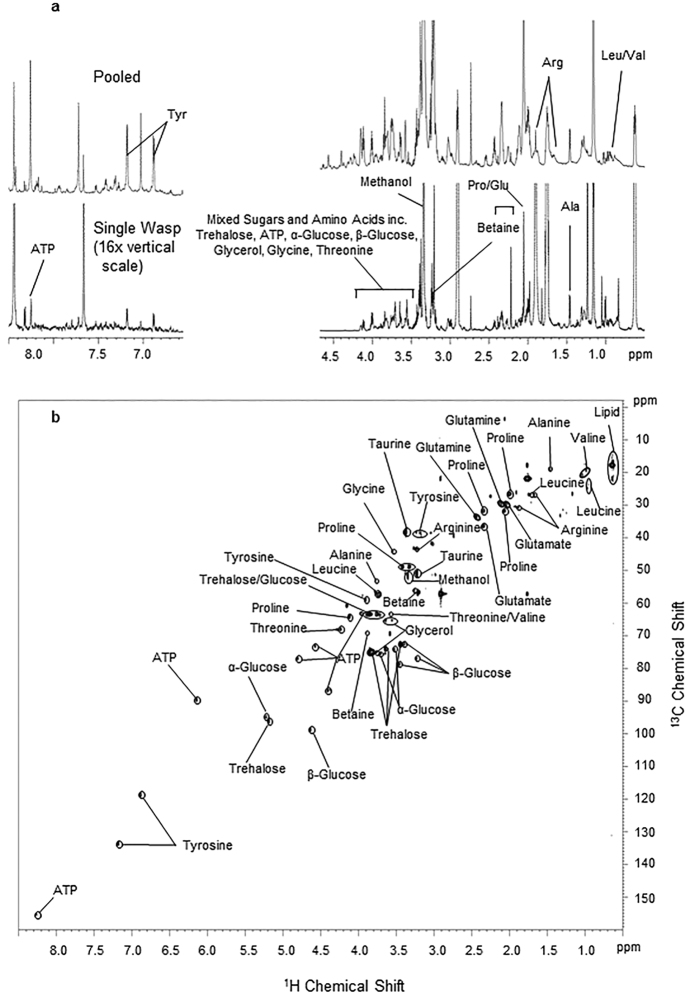
Representative ^1^H NMR and 2D spectrum of *Goniozus legneri* methanol extracts. (**a**) A spectral comparison between ^1^H NMR spectra of 20 wasps (pooled) and a single wasp amplified vertically ×16 with 1D spectral resonance assignments (**b**) 2D HSQC spectra of pooled wasp sample including NMR resonance assignments. Complete ^1^H and ^13^C chemical shift assignments are listed in Table 1.

NMR analysis of individual wasp extracts successfully generated complex high resolution spectra for which compound assignment was readily possible. A total of 19 unique compounds were verified; these included organic acids, free amino acids and assorted sugars (Fig. 1a). As with most biological samples, characterisation of some specific spectral resonances was not possible. A degree of overlap from major resonances in 1D spectra presented difficulties in initial assignment; this was a particular issue with the highly overlapping sugar signals in the region δ4–3 (Fig. 1a). These overlapping spectra were clearly assignable through analysis of 2D heteronuclear single quantum coherence (HSQC) spectra (Fig. 1b). 2D NMR peak assignments are further outlined in Table 1. The clustering of quality control (QC) stability replicates was also assessed to determine if any time-dependent changes in metabolite profile were present. Individual stability replicates clustered loosely together, remaining within their respective experimental treatments. Whilst some variation in sample positions was detected, there was no trend with time. With the exception of bin 8.53 (ATP), the peak area relative standard deviation (RSD) of all 10 key bins were within the acceptable threshold limits (<30%) (Supplementary Table S1). Specific key bin area (RSD) for QC samples were within the range 3.71 to 27.83% (see also supplementary Table S1).

### Metabolite profiling using LC-MS

**Figure 2:**
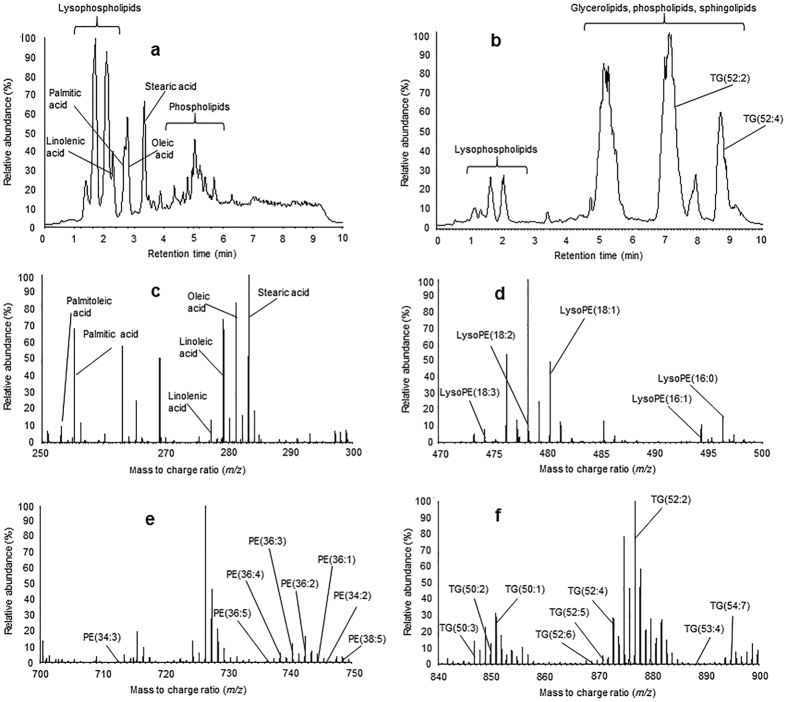
Representative total ion chromatograph of whole wasp extract in. (**a**) ESI- and (**b**) ES^+^ mode. Labels represent visible stability ions and major lipid categories (**C–E**) Representative mass spectra labelled for (**c**) fatty acid (**d**) lysophospholipid (**e**) phospholipid and (**f**) triacylglyceride key ions. PE = phosphoethanolamine, TG = triacylglyceride. The unusual appearance of lysophospholipids alongside fatty acids in ESI- mode was found to be due to in source fragmentation of ES + lysophospholipids. These peaks matched the retention time and corresponded to major LC-MS/MS fragments of the corresponding lysophospholipid seen in ESI+. These ions were absent from chromatograms acquired during Velos LC-MS/MS analysis.

**Figure 3:**
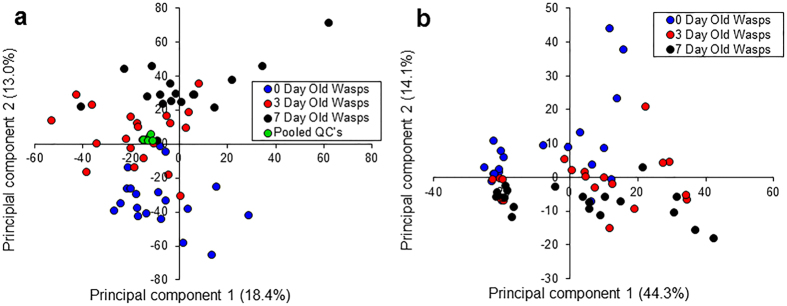
Principal components analysis of single wasp extracts. (**a**) PCA of LC-MS samples of wasps aged 0 days, 3 days and 7 days (PC1 = 18.4%, PC2 = 13.0%, PC3 = 10.5%) including pooled QC samples (**b**) PCA of NMR samples of wasps aged 0 days, 3 days and 7 days (PC1 = 44.3%, PC2 = 14.1%, PC3 = 8.28%).

Our LC-MS approach detected 1076 unique ions in an individual wasp extract. These comprised 360 ions in negative mode ESI and 749 in positive mode ESI. Lipid categories tentatively identified included fatty acids, phospholipids, sphingolipids, glycerolipids and lysophospholipids. ESI- mode spectra were dominated by common fatty acids and phospholipids (Fig. 2a). Spectra of ESI^+^ mode were dominated by large storage lipids and mobilisation lipids including diacyl- and triacylglycerides (Fig. 2b). QC samples exhibited very little drift over time in PCA comparisons (Fig. 3a), clustering centrally between experimental groups. Assessment of peak areas of key ions (Fig. 2c,f) within QC samples found that 32 out of 34 (94.12%) QC ions displayed RSDs within an arbitrarily defined, but widely accepted, acceptability threshold (<30%) for experimental stability. Assessment of retention time RSDs within QC samples found that all 34 QC ions displayed RSDs within the acceptability threshold for experimental stability. QC ions had an average peak area RSD of 10.29% with a range of 2.38–76.58% and an average retention time RSD of 0.9% with a range of 0.13–4.40%. These results confirm the validity and stability of the LC-MS analysis. Specific key ion peak area and retention time RSDs for both technical replicates and QC samples are provided in the Supplementary Table S2).

### Metabolite profiles in relation to age using NMR

**Figure 4:**
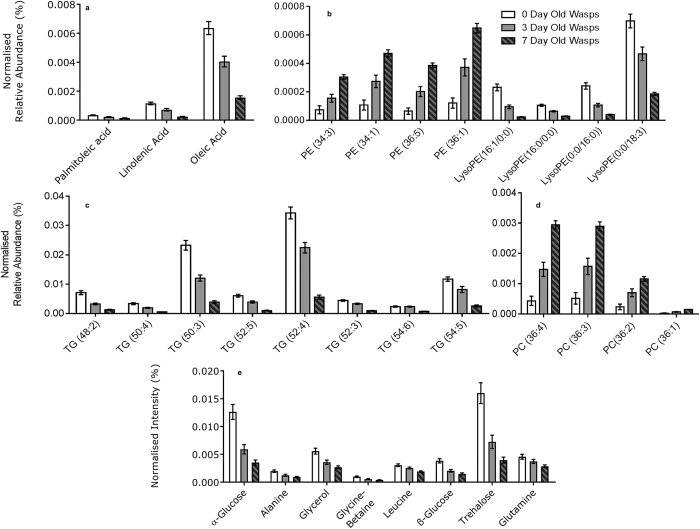
Metabolite differences between 1 day, 3 day and 7 day old *G. legneri*. Differential metabolites are separated by class and scale, comprising of (**a**) fatty acids (**b**) phosphoethanolamines (PE) and lysoPEs (**c**) triglycerides (**d**) phosphocholines (**e**) Sugars and amino acids. The displayed values consist of the mean normalised metabolite area; error bars show standard error.

Using NMR, the generated principal component analysis (PCA) model exhibited a goodness of fit (R^2^X) of 0.731 for wasp age treatments (0 days, 3 days and 7 days). Associated PCA scores plots indicated a degree of separation between 0 days and 7 days treatment, with 3 days samples indicating a loose degree of intermediate clustering (Fig. 3b). Further validation of weighted scores plots by Kruskal-Wallis tests generated a total of 80 significant bins between 0 day and 7 day old wasps. Of these bins, 73 were reduced in the 7 days treatment, compared to seven that were elevated. Spectral assignments of bins identified a number of metabolites that were reduced in 7 day old wasps, including common insect sugars and amino acids. Individual metabolite identities, *H* values and p-values are given in Table 2. Individual metabolite levels are displayed in Fig. 4.

### Metabolite profiles in relation to age using LC-MS

Using LC-MS, the generated PCA model for wasp age treatments exhibited an R^2^X of 0.833. Analysis of generated scores plots according to wasp ages indicated separation between 0 day old wasps and 7 day old wasps across the second principal component, whereas 3 day old wasps exhibited partial separation from and, clustered intermediately between the two (Fig. 3a). Comparisons between samples from 3 day old wasps and each of the other treatments found no clear separation. Assessment of potential biomarkers of ageing by Kruskal-Wallis tests resulted in a total of 59 unique ions successfully measured by LC-MS/MS that differed significantly between 0 day old and 7 day old wasps. These identifications comprised of 3 free fatty acids, 16 glycerolipids, and 40 phospholipids. All 16 glycerolipids were triacylglycerides, the primary form of storage in the insect fat body. All of these lipids were reduced in 7 day wasps compared to 0 day wasps, with 3 day old wasps exhibiting peak area levels intermediate between the two (Fig. 4). Differential phospholipids comprised of glycerophospholipids (23) and lysophospholipids (17). While lysophospholipids were reduced in 7 day old wasps, the majority of glycerophospholipids were elevated in 7 day old wasps (22 *vs*. 1 phosphocholine which was reduced). Individual mass-to-charge ratios, identities, elution times, adducts, *H* values and fold changes of the most significant non-polar metabolites are given in Table 3. The entire list of 59 differential non-polar metabolites can be found in Supplementary Table S3 online. Metabolite levels for each lipid category are displayed in Fig. 4. Representative MS/MS fragmentation spectra to confirm lipid classes are provided in the Supplementary Figure S2.

## Discussion

### Analytical method validation

This study aimed to address the challenges associated with employing a rigorous analytical approach to assess metabolomic changes in individual insects. Our approach further aimed to be efficient, precise and reproducible when being applied for the potential elucidation of experimental biomarkers^38^,^39^. Analysis of key bins/ions within NMR and LC-MS technical replicates indicated that the vast majority of peak area and retention time RSDs were well within the acceptability threshold of 30% for analytical precision (FDA USA^38^). Our approach was further demonstrated to be stable throughout the entire analytical timeframe, as more than 95% of key ions/bins were within the acceptability threshold for LC-MS and NMR stability samples respectively.

This developed analytical protocol successfully generated metabolite profiles of individual adult female *G. legneri* using both NMR and LC-MS approaches and demonstrated that these vary according to wasp age. This study further confirms that a two-step methanol/chloroform/water extraction protocol is suitable for obtaining metabolome profiles of small (approx. 1 mg) individual insects^37^,^40^. Furthermore, our protocol is capable of generating high resolution NMR and MS spectra that exhibit a high degree of conservation with previously reported insect spectral features^3^,^41^. More importantly, the protocol we present is capable of differentiating between polar metabolite profiles generated from individual wasps of different ages, despite their low biomass.

Moreover, our study shows that utilising high-field NMR and low-temperature probe technology we are able to characterise low concentration metabolites with estimates of detection at below the 1 nmol range (as estimated based on the intensity of our standards recorded in these conditions).

### The effects of aging on the *G. legneri* metabolome

Comparisons of NMR spectra between 0 day old and 7 day old wasps indicated a general decline of carbohydrates such as glucose, glycerol and trehalose (the latter being the most abundant carbohydrate of insect haemolymph) with age, indicating the loss of energy reserves expected from prolonged starvation. Furthermore, the decline of several amino acids after seven days can be viewed as the result of starvation, rather than the result of a temporary immediate decline due to cuticle sclerotization, as shown in other insects (e.g. mosquitoes^42^). The main class of metabolites that were reduced in 7 day old wasps were the triglycerides; these, along with glycogen, are considered the main energy reserves in animal cells and are the main constituent of the insect fat body (90%). In insects they are generally synthesized from dietary carbohydrates via lipogenesis^43^. However, parasitoids lack the ability to synthesize lipids from dietary intake, a characteristic considered an evolutionary result of their parasitic life-history^36^. Lipids, and their allocation to eggs and metabolic maintenance, have been studied holistically in insect parasitoids^44^. Our results show a gradual decline in triglyceride levels, consistent with reported patterns of fat reserve decline in starved parasitoids^44^,^45^. The similar levels of diglycerides observed across wasps of different ages is somewhat unexpected; diglycerides are considered the immediate breakdown products of triglycerides and also indicative of fat mobilisation in insect haemolymph^43^. Thus we could expect that temporary starvation would induce elevation in their levels, although prolonged starvation would lead to their ultimate depletion. Nonetheless, their levels were largely similar among age treatments. Lysophospholipids are important signalling molecules^46^ whilst glycerophospholipids are structural components in cell membranes^47^. Whereas the former declined in abundance in older parasitoids, the latter were elevated, probably as result of cellular membrane breakdown within the insect fat body.

Determining the age of wild-caught insects is an important tool for understanding their ecology, as lifespan typically correlates with reproductive success (≈evolutionary fitness). An estimate of age would be useful for understanding demographic trends^48^ and also behavioural decisions by insects in the field^49^,^50^. For parasitoids, such as the *Goniozus* wasps examined here, such information could indicate how habitat variables (e.g., floral resource provisioning) influence parasitoid efficiency in biological control programs^51^. Furthermore, assessing the fitness (both in terms of age and energetic reserves) of laboratory-reared genetically-modified mosquitoes that block malaria parasites, and of sterile males of the species that are released to suppress pest population growth, is mostly restricted to laboratory conditions that are rarely validated in the wild^52^. Our method could be further adapted to generate information on the aging and physiological status of both laboratory and field-caught individual insects. However, there are potential confounding factors in our experimental design that would have to be overcome in order to generate biologically relevant data for field investigations, in particular the variable nature of wild-caught insect diets. The NMR approach outlined here is presently not capable of distinguishing between an insect’s metabolome and resonances that are only present in the organism’s gastrointestinal tract. To circumvent this, no diet was provided to *G. legneri* throughout our experiment. This is not an important confounding factor in our study; as *G. legneri* lacks the ability to generate new energy stores after adult eclosion, gradual lipid loss due to starvation is an unavoidable part of the aging process for this particular organism. However, for non-parasitoids dietary variation (and restriction) can significantly alter the metabolomics of aging^53^ and should be taken into account in future studies.

In conclusion, we have demonstrated that a combined NMR and LC-MS based metabolomics approach is capable of producing high resolution spectra from single insects of low biomass. This approach also successfully detected age-related changes in the *G. legneri* metabolome, confirming that this tailored approach is capable of accurately detecting biological changes in low-biomass organisms. Compounds associated with energy metabolism were found to deteriorate with age, including haemolymph rich sugars and large storage glycerolipids, both of which are likely to be important influences on behaviour and ecological performance.

## Significance

Metabolomics studies of small organisms, such as insects, have hitherto relied on pooling samples due to detection limits, particularly those of NMR. In this study we show, for the first time, that untargeted profiling of both polar and non-polar metabolites, implementing a combined NMR and LC-MS approach, can be used reliably on individual minute (~1 mg) insects. Insects have played a considerable role in the study of animal behaviour and ecology and empirical investigations chiefly involve the observation of individuals. Further illustration of our approach is provided by the differential metabolite profiles of young and old individual parasitoid wasps, which for NMR is to our knowledge the first study reporting this approach for such a small organism. Lipids are a promising group of differential metabolites for the development of parasitoid age biomarkers because most parasitoid wasps lack lipogenesis. Moreover, determination of the age of field-caught individuals can be important in understanding their ecology and behaviour and thus can also generate improvements to ecosystem services, such as biological pest control and pollination, provided by studied species of parasitoids and other insects. Thus, approaches for assessing the metabolomes of individual, low-biomass, organisms are an important advance. This study not only forms a paradigm for addressing the practical obstacles faced in metabolomics studies, and particularly NMR, when biological material is limited but also provides the vanguard of the integration of metabolomics into behavioural and ecological studies, particularly as many studied species are of minute size.

## Methods

### Materials

Insect culturing materials: glycerol, honey, corn meal wheat bran and yeast.

Chemicals used: methanol, chloroform, water, isopropanol, acetonitrile, ammonium acetate

Mobile phases: (A) 80:10:10 water/isopropanol/acetonitrile modified with 0.01% ammonium acetate (B) 50:50 isopropanol/acetonitrile modified with 0.01% ammonium acetate.

All solvents used were of high LC-MS grade purity (Chromasolv, Sigma-Aldrich, United States).

### Parasitoid Rearing Protocol and Experimentation

The host of *G. legneri*, the rice moth *Corcyra cephalonica* (Stainton) (Lepidoptera: Pyralidae), was reared on the artificial laboratory diet outlined by Lizé *et al*.^35^. The parasitoid strain was originally obtained from a commercial insectary in the USA, and both host and parasitoid strains were the same as used in several previous behavioural studies^34^,^35^. *Goniozus legneri* were reared on larvae of *C. cephalonica* by introducing an adult female wasp and a caterpillar in a glass vial (2.5 × 7.5 cm) plugged by gauze and cotton. Parasitoid and moth culturing and all experiments were carried out in a climate room (~27 °C, continuous illumination, 60–70% relative humidity).

Individual *Goniozus legneri* females were presented with a *C. cephalonica* larva and upon emergence, their female offspring were weighed to an accuracy of 0.01 mg. All wasps used in experiments were within the range 0.94–1.63 mg. To assess the effects of age on metabolomic state, wasps were either sampled upon adult emergence (0 days) or placed in a 0.5 ml Eppendorf tube and subsequently sampled at three or seven days post-emergence.

### Metabolite extraction

Individual parasitoids were snap frozen in liquid N_2_ immediately after weighing, then stored at −80 °C until extraction. We used a modified Bligh & Dyer^37^ biphasic lipid extraction protocol with the final ratio of solvents used in a methanol/chloroform/water solution being 2.0:2.0:1.8; a two-step solvent addition process as described by Wu *et al*.^40^ was employed. All solvents were kept ice-cold throughout sample extraction. Single wasps were placed in a 2.0 ml homogenisation vial (lysing matrix Z MP-BIO^®^) and homogenised in 320 μl methanol and 128 μl water in a MP-BIO Fast Prep^®^ homogenizer (MP Biomedicals, United States). 320 μl of chloroform and 160 μl of water were then added and extracts were vortexed for 30 s. Extracts were then centrifuged for 10 min at 10,000 g and left on ice for 5 min to facilitate phase separation. For each extract, lipid phase (lower layer) was transferred to a 2 ml borosilicate glass vial. The upper phase (polar compounds, ~400 μl) were transferred to a 1.5 ml solvent resistant Eppendorf ^®^Biopur^®^ tube. Both phases were stored at −80 °C prior to analysis. Sample order was randomised prior to extraction and again prior to analysis to minimise unwanted variation and allow for the greatest reliability and validity of statistical estimates of treatment effects.

### Metabolite Profiling using NMR

Individual polar extracts were dried in a rotary evaporator and reconstituted in 600 μl of D_2_O with 0.5 mM of DSS (4,4-dimethyl-4-silapentane-1-sulfonic acid) added as internal reference prior to analysis. All NMR experiments were acquired on a Bruker Avance 800 MHz Avance III spectrometer using a 5 mm QCI Cryoprobe. Data were collected with a spectral width of 13 ppm and signal averaged over 512 scans using a noesy 1D pre-saturation experiment to achieve water suppression. Total recycling delay was 4.7 s. Pre-saturation power and frequency were determined in advance on representative samples and fixed for all acquisitions. Samples were referenced and locked using the internal D_2_O signal with a secondary reference of DSS (0.5 mM) present in all samples. Data were processed by application of an exponential window function set to give a 0.3 Hz line broadening prior to zero filling and Fourier transformation. To aid confirmation of metabolite identity, two-dimensional NMR spectra were generated from a pooled extract of 20 wasps generated utilising the same method. A heteronuclear single-quantum correlation (HSQC) experiment was employed. HSQC spectra were acquired to give sufficient signal-to-noise for analysis, depending on sample, this was between 40–160 transients and with between 400–512 points in t1 and 4096 complex points in t2 covering a ^1^H spectral width of 8484 Hz and a 13C spectral width of 33339 Hz. Throughout NMR analysis, spectra were acquired from 3 randomly selected samples from each treatment class after every ten spectral acquisitions. Stability sample replicates were assessed for clustering and drift over time. Ten metabolite associated bins were selected within each set of replicates and the relative standard deviations of their intensity were assessed.

### Metabolite profiling using LC-MS

Individual non-polar extracts were dried in a rotary evaporator and reconstituted in 100 μl of isopropanol prior to analysis. Accurate mass LC-MS was performed on lipid extracts (10 μl injection volume) of individual wasps using an Accela LC coupled with an Exactive mass spectrometer (ThermoFisher Scientific, USA) in positive and negative electrospray ionisation modes (ESI). An Ace Excel 2 Super C18 (2 μm particle size, 2.1 × 50 mm) column equipped with an appropriate guard column was maintained at 40 °C with a variable flow rate throughout analysis. The LC gradient program (total duration 10 min) had an initial starting proportion of 62.5% B with a flow rate of 300 μl followed by a linear increase to 99% B with a flow rate of 400 μl over 3 min. This proportion was maintained for a further 5 min followed by a linear decrease to the starting proportion of 62.5% B and flow rate of 300 μl over 2 min. Ions were monitored within the range of *m*/*z* 100 to 1500 (ESI voltage: 3500, capillary temperature: 350 °C, scan rate: 250 ms, FT resolution: 25,000). A pooled quality control sample comprising 10 μL from each experimental sample was generated and injected throughout the run. Stability sample replicates were assessed for clustering and drift over time. A set of key ions were selected within each set of replicates and the RSDs of their peak area and retention times.

### Metabolite identification by MS/MS analysis

A tandem mass spectrometry approach was employed to validate putative identifications of differential ions. Remaining lipid extracts were pooled prior to analysis to improve yield. Analysis was performed utilising an Accela LC coupled with an LTQ Velos Pro Dual-Pressure linear ion trap mass spectrometer (Thermo Fisher Scientific, USA). The column, mobile phases and gradient programs were replicated from the previous LC-MS analysis. Ions were monitored within the range of *m*/*z* 100 to 1500 (ESI voltage: 3000, capillary temperature: 275 °C, scan rate: 50 ms). MS2 fragmentations were performed under a collision energy of 40 V with a maximum ion isolation window of 5 Da.

### Data processing and statistical analysis

All NMR spectra were referenced to the internal DSS signal at 0 ppm. Individual spectra were manually phased and baseline corrected in Topspin (Bruker Scientific Instruments, Bruker) prior to non-discriminant data alignment and binning utilising user generated scripts and in-build library calls to determine the area under the curve. Data simplification was achieved by dividing spectra into a total of 931 non-overlapping regions (bins) with a width of 0.01 ppm, to which integrated spectral intensities were assigned. The water signal region (5.2–4.5) was excluded prior to multivariate analysis.

LC-MS data were aligned and framed without discrimination using the propriety software Sieve 2.0 (Thermofisher Scientific). A frame width of 0.5 min was employed during alignment to remove variation caused by minor chromatographic shifts. Samples were framed with an arbitrary intensity threshold of 50,000. The first 0.5 min of spectral acquisition were excluded from framing to remove variation from potential polar metabolite contamination.

The generated frame/bin tables for both NMR and LC-MS were exported to a spreadsheet package (Microsoft Excel) and normalised to total ion intensity to account for small variations in sample concentration. Normalised tables were separately imported into the Simca + (Umetrics) package, where preliminary sample classification was visualised using a Principal Components Analysis (PCA). Individual variables were mean-centred and autoscaled prior to model fitting. Comparisons of the generated PCA loading and scores plots were utilised to aid in the selection of potential biomarkers, along with the generation of class weightings for each variate. Differential ions were validated with a Kruskal-Wallis test using the statistical package GenStat (v.15, VSN International), specifying ion count normalised to total sample intensity as the response variable. Generated p-values were adjusted using the Bonferroni correction to account for multiple hypothesis testing^16^. Metabolites with an adjusted p-value <0.05 were considered to be validated as differential.

Putative NMR resonances were validated through direct comparisons between generated 2D NMR spectra and 2D spectral standards from the Biological Magnetic Resonance Data Bank (BMRB). Individual standards were aligned to the D_2_O signal in topspin then validated by overlaying them directly with the 2D wasp spectra using CCPNMR (CCPN project). Putative LC-MS ion identifications were validated through direct comparisons of generated LC-MS/MS fragmentation spectra with the ‘Lipidblast fragmentation database’^54^. Further validation was provided by direct comparisons to external lipid standard databases (Lipidmaps, HMDB, Massbank databases).

The research procedures undertaken here using insects are accordance with the principles of Replacement, Reduction and Refinement as set by ARRIVE (Animal Research: Reporting of *In Vivo* Experiments) guidelines and enforced by the University of Nottingham Animal Welfare and Ethical Review Body (AWERB).

## Additional Information

**How to cite this article**: Kapranas, A. *et al*. Metabolomics of aging assessed in individual parasitoid wasps. *Sci. Rep*. **6**, 34848; doi: 10.1038/srep34848 (2016).

## Supplementary Material

Supplementary Information

## Figures and Tables

**Table 1:** ^1^H and ^13^C chemical shift assignments of *G. legneri* NMR spectra.

Metabolite	^1^H Chemical Shift (Multiplicity^a^)	^13^C Chemical Shift
ATP	4.24 (m), 4.30 (m), 4.41 (m), 4.58 (m), 4.77 (t), 6.15 (d), 8.26 (s), 8.53 (s)	67.78, 67.78, 86.68, 72.96, 72.96, 89.57, 155.42, 142.76
Formate	8.45 (s)	—
Tyrosine	3.04 (dd), 3.18 (dd), 3.92 (dd), 6.89 (d), 7.19 (d)	38.58, 38.51, 58.94, 118.68, 133.52
α-Glucose	3.45 (t), 3.52 (dd), 3.70 (t), 3.76 (m), 3.82 (ddd), 3.83 (m), 5.22 (d)	72.42, 74.2, 75.53, 63.34, 74.15, 63.34, 94.86
Trehalose	3.44 (t), 3.64 (dd), 3.75 (m), 3.82 (m), 3.84 (m), 3.86 (m), 5.19 (d)	72.47, 73.82, 63.32, 74.93, 75.30, 63.33, 96.02
β-Glucose	3.24 (dd), 3.4 (m), 3.47 (m), 3.74 (m), 3.88 (dd), 4.64 (d)	76.8, 72.4, 78.65, 63.28, 63.28, 98.67
Threonine	1.32 (d), 3.59 (d), 4.24 (m)	63.21, 67.98
Proline	2.01 (m), 2.08 (m), 2.35 (m), 3.33 (dt), 3.42 (dt), 4.12 (dd)	26.5, 31.66, 31.73, 48.84, 48.84, 64.06
Glycine-Betaine	3.26 (s), 3.90 (s)	56.16, 68.93
Arginine	1.67 (m), 1.91 (m), 3.23 (t), 3.76 (t)	26.72, 30.42, 43.33, 57.05
Alanine	1.47 (d), 3.77 (q)	18.99
Glycerol	3.55 (m), 3.64 (m), 3.77 (tt)	65.24, 65.24, 74.94
Glutamine	2.12 (m), 2.44 (m), 3.76 (t)	29.57, 33.61, 56.96
Glutamate	2.05 (m) 2.12 (m), 2.34 (m), 3.74 (d)	29.72, 29.72, 36.35, 57.37
Valine	0.99 (d), 1.04 (d), 2.34 (m), 3.6 (d)	19.41, 20.73, 31.66, 63.04
Glycine	3.54 (s)	44.23
Taurine	3.23 (t), 3.39 (t)	50.78, 38.24
Methanol	3.35 (s)	51.66
Leucine	0.95 (t), 1.75 (m), 3.74 (m)	23.6, 26.62, 56.86

Letters m, t, d, s & q stand for peak multiplicity, where m = multiplet, t = triplet, d = doublet, s = singlet & q = quartet. The presence of methanol resonances is likely due to minor contamination from the extraction process.

**Table 2:** Summary of polar biomarkers with tentative identities that significantly differ between 0 day old and 7 day old wasp extracts.

Common Name	Chemical shift (ppm)	d.f.	Formula	*H* value	p-value	Fold change
α-Glucose	3.45 (t), 3.52 (dd), 3.70 (t), 3.76 (m), 3.82 (ddd), 3.83 (m), 5.22 (d)	2	C_6_H_12_O_6_	34.92	<0.01	−3.58
Alanine	1.47 (d), 3.77 (q)	2	C_3_H_7_NO_2_	17.18	<0.01	−2.11
Glycerol	3.55 (m), 3.64 (m), 3.77 (tt)	2	C_3_H_8_O_3_	16.47	<0.01	−2.08
Glycine-Betaine	3.26 (s), 3.90 (s)	2	C_5_H_11_NO_2_	25.60	<0.01	−2.53
Leucine	0.95 (t), 1.75 (m), 3.74 (m)	2	C_6_H_13_NO_2_	16.53	<0.01	−1.59
ß-Glucose	3.24 (dd), 3.4 (m), 3.47 (m), 3.74 (m), 3.88 (dd), 4.64 (d)	2	C_6_H_12_O_6_	29.35	<0.01	−2.57
Trehalose	3.44 (t), 3.64 (dd), 3.75 (m), 3.82 (m), 3.84 (m), 3.86 (m), 5.19 (d)	2	C_12_H_22_O_11_	34.79	<0.01	−4.04
Glutamine	2.12 (m), 2.44 (m), 3.76 (t)	2	C_5_H_10_N_2_O_3_	11.18	<0.05	−1.61

Fold changes were detected with Kruskal-Wallis tests (non-parametric). Negative and positive fold changes indicate metabolites that declined and increased respectively in abundance between 0 day old and 7 day old wasps.

**Table 3:** Representative non-polar biomarkers with tentative identities that significantly differ between 0 day old and 7 day old wasp extracts.

*M/Z*	Average RT (min)	Adduct	ESI Phase	Common Name	Formula	d.f.	*H* value	p-value	Fold change	Mass error (ppm)
450.263	1.62	M–H	Negative	LysoPE(16:1/0:0)	C_21_H_42_NO_7_P	2	30.46	<0.01	−10.13	0.387
452.277	1.58	M + H	Positive	LysoPE(0:0/16:0)	C_21_H_44_NO_7_P	2	35.43	<0.01	−6.08	0.737
494.324	1.50	M + H, M + NA	Positive	LysoPC(0.0/16.1)	C_24_H_48_NO_7_P	2	27.73	<0.01	−5.98	0.115
682.448	6.16	M + Na	Positive	PE(30:2)	C_35_H_66_NO_8_P	2	31.57	<0.01	6.78	0.8
736.493	4.13	M-H	Negative	PE(36:5)	C_41_H_72_NO_7_P	2	25.10	<0.01	6.01	0.721
738.509	4.38	M-H	Negative	PE(36:4)	C_41_H_74_NO_8_P	2	20.74	<0.05	6.74	1.071
740.521	4.32	M + H	Positive	PE(36:4)	C_41_H_74_NO_8_P	2	34.98	<0.01	6.59	0.171
762.509	4.31	M-Hac-H	Negative	PC(30:1)	C_38_H_74_NO_8_P	2	31.37	<0.01	6.85	3.479
782.568	4.32	M + H	Positive	PC(34:1)	C_42_H_82_NO_8_P	2	32.55	<0.01	6.44	0.977
792.707	7.83	M + NH4	Positive	TG(42:2(12:0/18.1/16.1)	C_45_H_82_O_6_	2	41.44	<0.01	−5.73	0.564
818.496	4.13	M-Hac-H	Negative	PC(34:1)	C_42_H_82_NO_8_P	2	33.07	<0.01	6.34	1.756
820.738	8.90	M + H, M + NA	Positive	TG(48:2(16:0/16.1/16.1)	C_51_H_94_O_6_	2	40.41	<0.01	−5.63	0.864
838.561	4.09	M-Hac-H	Negative	PC(36:5)	C_44_H_78_NO_7_P	2	32.19	<0.01	6.35	0.644
840.576	4.38	M-Hac-H	Negative	PC(36:4)	C_44_H_80_NO_7_P	2	33.16	<0.01	6.74	0.006
844.738	7.90	M + NH4	Positive	TG(50:4(16:0/16.1/18.3)	C_53_H_96_O_5_	2	38.61	<0.01	−6.56	0.864
846.753	8.71	M + H, M + NA	Positive	TG(50:3(16:0/16.1/18.2)	C_54_H_98_O_6_	2	42.76	<0.01	−5.99	1.514
868.738	7.22	M + NH4	Positive	TG(52:6(16:1/18.2/18.3)	C_55_H_94_O_6_	2	27.62	<0.01	−18.77	0.864
870.753	7.90	M + NH4	Positive	TG(52:5(16:0/18.2/18.3)	C_55_H_98_O_5_	2	35.56	<0.01	−6.05	0.894
872.769	8.74	M + H, M + NA	Positive	TG(52:4(16:0/18.2/18.2)	C_55_H_98_O_6_	2	39.42	<0.01	−6.13	1.164
876.799	8.67	M + NH4	Positive	TG(52:2(16:0/18.1/18.1)	C_55_H_102_O_6_	2	31.34	<0.01	−7.43	2.464

PE = phosphoethanolamine, PC = phosphocholine. Fold changes were detected with Kruskal-Wallis tests (non-parametric). Negative and positive fold changes indicate metabolites that declined and increased respectively in abundance between 0 day old and 7 day old wasps.
