# Supplementary material for: Metabolomics of aging assessed in individual parasitoid wasps
Source: Sci Rep. 2016 Oct 7;6:34848. doi: 10.1038/srep34848 (PMC5054366; doi:10.1038/srep34848)
Supplement: Supplementary Information [file srep34848-s1.pdf]

Metabolomics of aging assessed in individual parasitoid wasps

Apostolos Kapranas, Charles J. P. Snart, Huw Williams, Ian C. W. Hardy & David A. Barrett

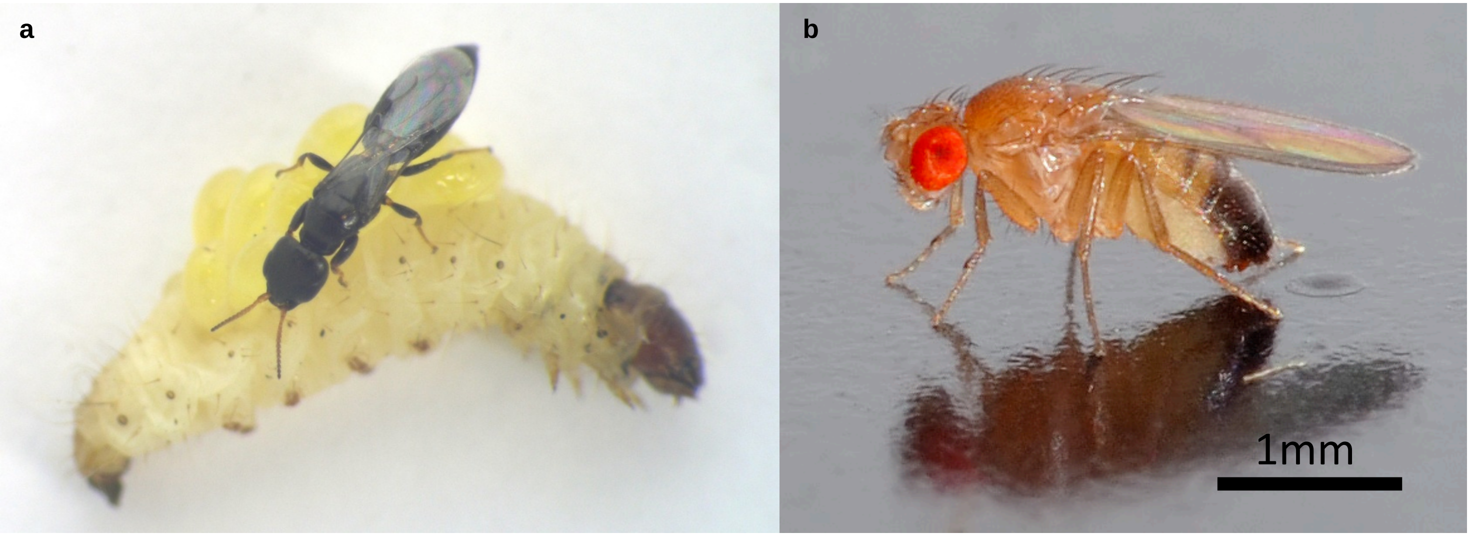

Supplementary Figure S1. Comparison of (a) *Goniozus legneri* and (b) *Drosophila melanogaster. G. legneri* is of a similar biomass to that of *D. melanogaster* (~1mg) Photos: (a) Sonia Dourlot (b) André Karwath, Wikimedia Commons. Photo is reproduced under the Creative Commons Attribution-Share Alike 2.5 Generic license.: https://creativecommons.org/licenses/by-sa/2.5/deed.en
https://en.wikipedia.org/wiki/Drosophila_melanogaster
https://upload.wikimedia.org/wikipedia/commons/4/4c/Drosophila_melanogaster_-_side_(aka).jpg

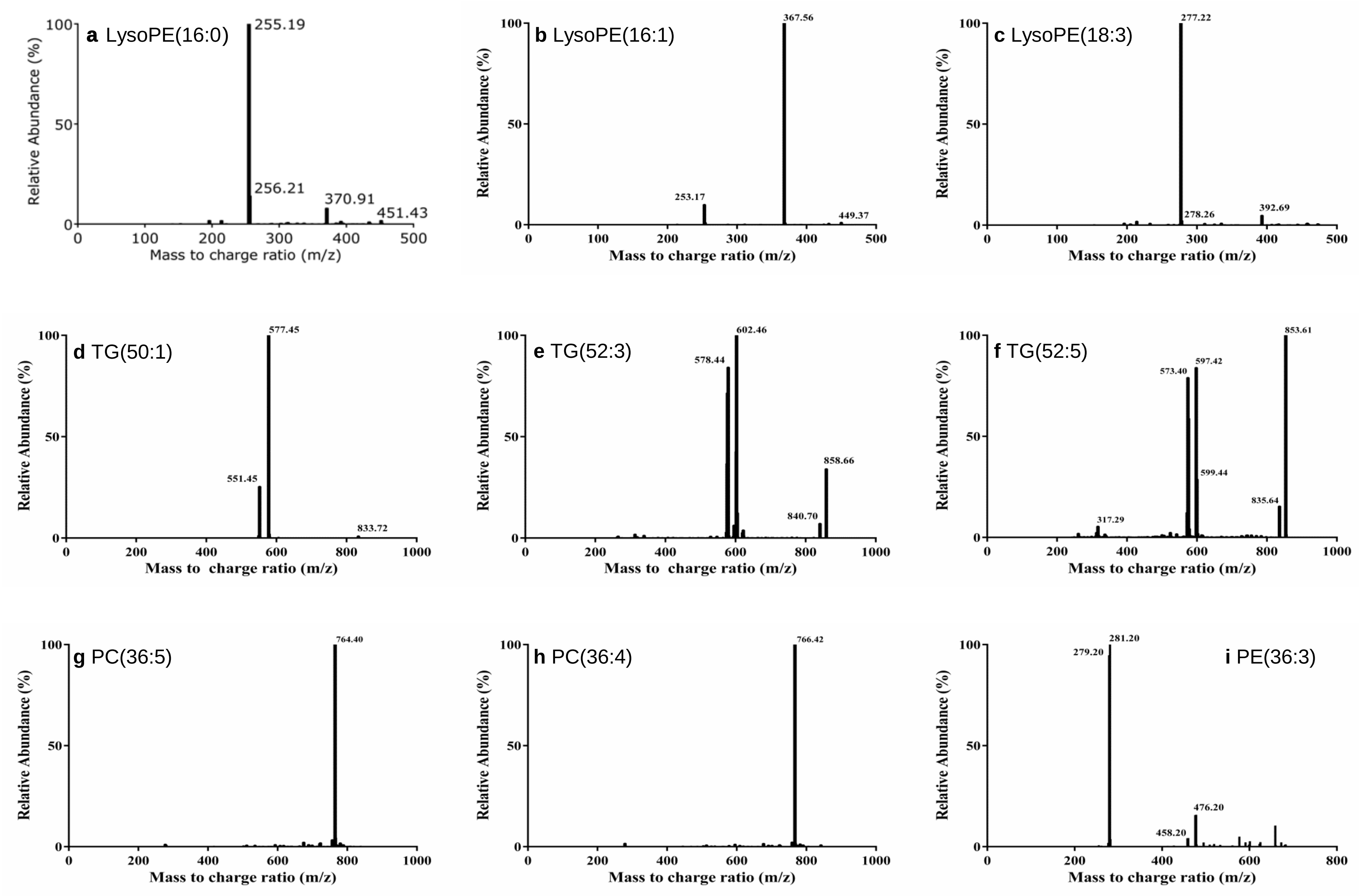

Supplementary Figure S2. LC-MS-MS spectra of differential lipid classes (a-c) Lipophospholipids (d-f) Triacylglycerides (g-i) Phospholipids.

Supplementary Table S1. Relative standard deviations of key spectral bins for NMR stability replicates (QC = quality control sample).

	**Relative Standard Deviation (%)**		
**Bin Identity (ppm)**	**QC1**	**QC2**	**QC3**
3.70 (Glucose)	3.71	22.62	7.86
8.53 (ATP)	27.83	41.58	27.15
3.82 (Trehalose)	6.90	23.61	14.77
2.01 (Arginine)	11.67	13.76	9.17
3.55 (Glycerol)	10.54	5.74	2.66
2.34 (Glutamate)	16.62	7.30	19.79
0.99 (Valine)	15.85	14.82	22.89
2.01 (Proline)	11.67	13.76	9.17
3.59 (Threonine)	11.78	5.01	4.85
0.95 (Leucine)	12.44	8.05	10.48

Supplementary Table S2. LC-MS key ion peak area RSDs (%) for technical replicates and quality control (QC) samples. RT = retention time, RSD = relative standard deviation. PE = phosphoethanolamine, PC = phosphocholine, PG = phosphoglycerol, DG = diacylglyceride, TG = triacylglyceride.

M/Z	**Average RT (min)**	**Tentative Identification**	**Phase**	**Adduct**	**Formula**	**Mass error (ppm)**	**Replicate peak area RSD (%)**	**Repliate peak area RSD range (%)**	**QC peak area RSD (%)**
253.217	2.13	Palmitoleic acid	Negative	M-H	$C_{16}H_{30}O_2$	0.073	5.32	0.58 - 13.73	6.24
255.233	2.67	Palmitic acid	Negative	M-H	$C_{16}H_{32}O_2$	0.03	3.88	1.34 - 12.12	26.07
277.218	1.91	Linolenic Acid	Negative	M-H	$C_{18}H_{30}O_2$	0.120	4.11	0.38 - 16.31	13.74
281.249	2.79	Oleic Acid	Negative	M-H	$C_{18}H_{34}O_2$	0.180	4.11	0.21 - 25.33	2.39
283.264	3.35	Stearic Acid	Negative	M-H	$C_{18}H_{36}O_2$	0.030	9.05	0.75 - 8.87	10.88
452.277	1.57	LysoPE(16:0)	Positive	M+H	$C_{21}H_{44}NO_7P$	0.189	5.16	0.04 - 9.76	3.59
474.267	0.95	LysoPE(18:3)	Positive	M+H	$C_{23}H_{42}NO_7P$	5.8	4.13	0.50 - 13.58	18.32
478.293	1.72	LysoPE(18:2)	Positive	M+H	$C_{23}H_{44}NO_7P$	0.2	3.33	0.17 - 9.51	2.69
480.308	2.13	LysoPE(18:1)	Positive	M+H	$C_{23}H_{46}NO_7P$	0.5	3.25	0.35 - 8.31	2.40
494.324	1.49	LysoPC(16.1)	Positive	M+H	$C_{24}H_{48}NO_7P$	0.139	9.88	0.23 - 8.24	4.45
496.342	1.95	LysoPC(16.0)	Positive	M+H	$C_{24}H_{50}NO_7P$	0.1	2.87	1.57 - 19.08	3.63
610.540	5.15	DG(34:2)	Positive	M+NH4	$C_{37}H_{68}O_5$	0.298	4.53	0.56 - 11.84	4.28
612.556	5.52	DG(34:1)	Positive	M+NH4	$C_{37}H_{70}O_5$	0.448	4.41	0.96 - 10.12	4.08
617.511	5.51	DG(36:4)	Positive	M+H	$C_{39}H_{68}O_5$	2.975	4.21	1.50 - 8.20	8.49
641.511	5.13	DG(36:3)	Positive	M+Na	$C_{39}H_{70}O_5$	0.743	4.35	0.55 - 9.17	7.16
643.526	5.52	DG(36:2)	Positive	M+Na	$C_{39}H_{72}O_5$	0.993	4.92	1.32 - 8.79	9.04
712.493	4.41	PE(34:3)	Negative	M-H	$C_{39}H_{72}NO_8P$	0.645	6.36	1.30 - 22.37	3.93
736.493	4.13	PE(36:5)	Negative	M-H	$C_{41}H_{72}NO_7P$	0.845	6.47	2.07 - 12.78	4.74
738.509	4.39	PE(36:4)	Nega tive	M-H	$C_{41}H_{74}NO_8P$	0.695	5.14	1.19 - 12.74	4.42
740.524	4.65	PE(36:3)	Negative	M-H	$C_{41}H_{76}NO_8P$	0.245	7.36	0.02 - 14.53	5.10
742.530	5.02	PE(36:2)	Negative	M-H	$C_{41}H_{78}NO_7P$	0.980	14.41	0.32 - 18.55	76.58
744.555	5.45	PE(36:1)	Negative	M-H	$C_{41}H_{80}NO_7P$	0.345	5.01	3.51 - 25.37	3.51
745.503	5.45	PG(34:2)	Negative	M-H	$C_{40}H_{75}O_{10}P$	0.715	8.45	0.33 - 11.36	12.17
748.529	4.39	PE(38:5)	Negative	M-H	$C_{43}H_{76}NO_7P$	0.260	17.73	1.15 - 20.09	5.13
794.496	5.08	PE(36:6)	Negative	M+Hac-H	$C_{41}H_{70}NO_8P$	1.756	5.07	2.12 - 34.44	9.88
846.753	8.71	TG(50:3)	Positive	M+NH4	$C_{54}H_{98}O_6$	1.214	2.04	0.21 - 6.18	5.11
849.772	5.29	TG(50:2)	Positive	M+NH4	$C_{53}H_{98}O_6$	2.236	3.49	0.81 - 4.62	6.04
850.784	7.29	TG(50:1)	Positive	M+NH4	$C_{53}H_{102}O_5$	1.614	4.35	1.12 - 7.78	3.73
868.738	8.71	TG(52:6)	Positive	M+NH4	$C_{55}H_{94}O_6$	1.364	3.78	0.32 - 12.09	4.83
870.753	7.95	TG(52:5)	Positive	M+NH4	$C_{55}H_{98}O_5$	1.814	3.21	0.38 - 8.47	5.84
872.769	8.73	TG(52:4)	Positive	M+NH4	$C_{55}H_{98}O_6$	1.464	3.89	0.23 - 7.20	5.04
876.781	7.09	TG(52:2)	Positive	M+NH4	$C_{55}H_{102}O_6$	0.564	4.53	0.07 - 42.60	39.63
887.775	7.48	TG(53:4)	Positive	M+NH4	$C_{56}H_{100}O_6$	7.735	4.85	0.76 - 7.76	20.99
894.754	8.73	TG(54:7)	Positive	M+NH4	$C_{57}H_{98}O_5$	0.114	4.61	0.77 - 12.20	5.93

SupplementaryTable S2 *cont*: LC-MS key ion retention time RSDs (%) for technical replicates and quality control (QC) samples. RT = retention time, RSD = relative standard deviation. PE = phosphoethanolamine, PC = phosphocholine, PG = phosphoglycerol, DG = diacylglyceride, TG = triacylglyceride.

M/Z	**Average RT (min)**	**Tentative identification**	**Phase**	**Adduct**	**Formula**	**Mass error (ppm)**	**RT RSD (%)**	**RT RSD range (%)**	**QC RT RSD (%)**
253.217	2.13	Palmitoleic acid	Negative	M-H	$C_{16}H_{30}O_2$	0.073	2.43	0.44 - 6.43	1.89
255.233	2.67	Palmitic acid	Negative	M-H	$C_{16}H_{32}O_2$	0.03	0.75	0.26 - 1.18	0.87
277.218	1.91	Linolenic Acid	Negative	M-H	$C_{18}H_{30}O_2$	0.120	1.03	0.15 - 2.26	1.66
281.249	2.79	Oleic Acid	Negative	M-H	$C_{18}H_{34}O_2$	0.180	1.26	0.04 - 6.04	1.09
283.264	3.35	Stearic Acid	Negative	M-H	$C_{18}H_{36}O_2$	0.030	0.60	0.17 - 1.08	0.70
452.277	1.57	LysoPE(16:0)	Positive	M+H	$C_{21}H_{44}NO_7P$	0.189	1.17	0.09 - 1.81	1.64
474.267	0.95	LysoPE(18:3)	Positive	M+H	$C_{23}H_{42}NO_7P$	5.8	2.01	0.14 - 3.75	2.96
478.293	1.72	LysoPE(18:2)	Positive	M+H	$C_{23}H_{44}NO_7P$	0.2	1.02	0.10 - 1.74	1.38
480.308	2.13	LysoPE(18:1)	Positive	M+H	$C_{23}H_{46}NO_7P$	0.5	1.04	0.39 - 1.64	1.24
494.324	1.49	LysoPC(16.1)	Positive	M+H	$C_{24}H_{48}NO_7P$	0.139	1.24	0.42 - 1.81	1.67
496.342	1.95	LysoPC(16.0)	Positive	M+H	$C_{24}H_{50}NO_7P$	0.1	1.98	0.60 - 3.53	2.71
610.540	5.15	DG(34:2)	Positive	M+NH4	$C_{37}H_{68}O_5$	0.298	0.43	0.04 - 0.72	0.56
612.556	5.52	DG(34:1)	Positive	M+NH4	$C_{37}H_{70}O_5$	0.448	0.44	0.06 - 0.76	0.50
617.511	5.51	DG(36:4)	Positive	M+H	$C_{39}H_{68}O_5$	2.975	0.43	0.06 - 0.74	0.46
641.511	5.13	DG(36:3)	Positive	M+Na	$C_{39}H_{70}O_5$	0.743	0.40	0.04 - 0.78	0.56
643.526	5.52	DG(36:2)	Positive	M+Na	$C_{39}H_{72}O_5$	0.993	0.47	0.06 - 0.95	0.50
712.493	4.41	PE(34:3)	Negative	M-H	$C_{39}H_{72}NO_8P$	0.645	1.46	0.02 - 2.35	2.76
736.493	4.13	PE(36:5)	Negative	M-H	$C_{41}H_{72}NO_7P$	0.845	0.58	0.18 - 0.99	0.96
738.509	4.39	PE(36:4)	Negative	M-H	$C_{41}H_{74}NO_8P$	0.695	0.95	0.26 - 1.64	1.64
740.524	4.65	PE(36:3)	Negative	M-H	$C_{41}H_{76}NO_8P$	0.245	0.65	0.03 - 2.37	2.41
742.530	5.02	PE(36:2)	Negative	M-H	$C_{41}H_{78}NO_7P$	0.980	1.15	0.13 - 3.98	4.52
744.555	5.45	PE(36:1)	Negative	M-H	$C_{41}H_{80}NO_7P$	0.345	1.14	0.20 - 2.05	2.04
745.503	5.45	PG(34:2)	Negative	M-H	$C_{40}H_{75}O_{10}P$	0.715	0.40	0.18 - 0.85	0.39
748.529	4.39	PE(38:5)	Negative	M-H	$C_{43}H_{76}NO_7P$	0.260	1.38	0.12 - 2.09	1.65
794.496	5.08	PE(36:6)	Negative	M+Hac-H	$C_{41}H_{70}NO_8P$	1.756	1.04	0.10 - 2.17	1.87
846.753	8.71	TG(50:3)	Positive	M+NH4	$C_{54}H_{98}O_6$	1.214	0.49	0.18 - 0.97	1.00
849.772	5.29	TG(50:2)	Positive	M+NH4	$C_{53}H_{98}O_6$	2.236	0.61	0.10 - 1.12	0.93
850.784	7.29	TG(50:1)	Positive	M+NH4	$C_{53}H_{102}O_5$	1.614	0.49	0.06 - 0.72	1.08
868.738	8.71	TG(52:6)	Positive	M+NH4	$C_{55}H_{94}O_6$	1.364	0.52	0.10 - 0.83	0.76
870.753	7.95	TG(52:5)	Positive	M+NH4	$C_{55}H_{98}O_5$	1.814	0.57	0.14 - 0.99	0.87
872.769	8.73	TG(52:4)	Positive	M+NH4	$C_{55}H_{98}O_6$	1.464	0.43	0.18 - 0.85	0.98
876.781	7.09	TG(52:2)	Positive	M+NH4	$C_{55}H_{102}O_6$	0.564	0.55	0.13 - 1.08	0.96
887.775	7.48	TG(52:0)	Positive	M+NH4	$C_{56}H_{100}O_6$	7.735	0.46	0.15 - 0.82	0.82
253.217	8.73	TG(54:7)	Positive	M+NH4	$C_{57}H_{98}O_5$	0.114	0.46	0.05 - 0.75	0.75

Supplementary Table 3: Summary of 59 non-polar biomarkers with tentative identities that significantly differ between 0 day old and 7 day old wasp extracts. PE = phosphoethanolamine, PC = phosphocholine. Fold changes were detected with Kruskal-Wallis tests (non-parametric). Negative and positive fold changes indicate metabolites that declined and increased respectively in abundance between 0 day old and 7 day old wasps

M/Z	Average RT (min)	Adduct	ESI Phase	Common Name	Formula	d.f.	*H* value	P value	Fold change	Mass error (ppm)
253.217	2.14	M-H	Negative	Palmitoleic acid	$C_{16}H_{30}O_2$	2	26.79	<0.01	-2.32	0.304
277.218	1.41	M-H	Negative	Linolenic Acid	$C_{18}H_{30}O_2$	2	31.65	<0.01	-4.77	0.696
281.249	2.13	M-H	Negative	Oleic Acid	$C_{18}H_{34}O_2$	2	17.86	<0.01	-4.09	0.396
450.263	1.62	M-H	Negative	LysoPE(16:1/0:0)	$C_{21}H_{42}NO_7P$	2	30.46	<0.01	-10.13	0.387
452.277	1.58	M+H	Positive	LysoPE(0:0/16:0)	$C_{21}H_{44}NO_7P$	2	35.43	<0.01	-6.08	0.737
452.279	2.05	M-H	Negative	LysoPE(16:0/0:0)	$C_{21}H_{44}NO_7P$	2	34.62	<0.01	-3.76	0.737
474.263	1.44	M-H	Negative	LysoPE(18:3/0:0)	$C_{23}H_{42}NO_7P$	2	35.79	<0.01	-4.69	0.387
476.277	1.43	M+H, M+NA	Positive	LysoPE(0:0/18:3)	$C_{23}H_{42}NO_7P$	2	31.34	<0.01	-3.77	1.263
476.279	1.74	M-H	Negative	LysoPE(18:2(9Z,12Z)/0:0)	$C_{23}H_{44}NO_7P$	2	29.86	<0.01	-4.44	0.737
478.293	1.75	M+H, M+NA	Positive	LysoPE(0:0/18:2)	$C_{23}H_{44}NO_7P$	2	30.38	<0.01	-3.25	0.087
478.294	2.13	M-H	Negative	LysoPE(18:1(9Z)/0:0)	$C_{23}H_{46}NO_7P$	2	28.94	<0.01	-4.05	0.087
480.308	2.17	M+H, M+NA	Positive	LysoPE(0:0/18:1)	$C_{23}H_{46}NO_7P$	2	29.73	<0.01	-3.10	0.465
494.324	1.50	M+H, M+NA	Positive	LysoPC(0.0/16.1)	$C_{24}H_{48}NO_7P$	2	27.73	<0.01	-5.98	0.115
496.34	1.96	M+H, M+NA	Positive	LysoPC(0.0/16.0)	$C_{24}H_{50}NO_7P$	2	34.56	<0.01	-2.99	0.235
506.325	2.05	M-H	Negative	LysoPE(20:1(11Z)/0:0)	$C_{25}H_{50}NO_7P$	2	27.25	<0.01	-3.72	0.213
520.339	1.35	M+H, M+NA	Positive	LysoPC(0:0/18:2)	$C_{26}H_{50}NO_7P$	2	31.57	<0.01	-3.67	0.765
522.355	2.09	M+H, M+NA	Positive	LysoPC(0:0/18:1)	$C_{26}H_{52}NO_7P$	2	29.85	<0.01	-3.73	0.416
540.305	1.35	M+Na	Positive	LysoPC(0:0/18:3)	$C_{26}H_{48}NO_7P$	2	31.67	<0.01	-4.26	1.057
546.352	2.64	M+Na	Positive	LysoPC(0:0/18:0)	$C_{26}H_{54}NO_7P$	2	29.62	<0.05	-1.79	1.008
580.363	2.04	M-Hac-H	Negative	LysoPC(18:1)	$C_{26}H_{52}NO_7P$	2	20.12	<0.01	-3.88	1.009
682.448	6.16	M+Na	Positive	PE(30:2)	$C_{35}H_{66}NO_8P$	2	31.57	<0.01	6.78	0.8
712.494	4.38	M-H	Negative	PE(34:3)	$C_{39}H_{72}NO_8P$	2	41.00	<0.01	4.12	1.721
716.525	5.03	M-H	Negative	PE(34:1)	$C_{39}H_{76}NO_8P$	2	25.61	<0.01	4.39	1.421
736.493	4.13	M-H	Negative	PE(36:5)	$C_{41}H_{72}NO_7P$	2	25.10	<0.01	6.01	0.721
738.509	4.38	M-H	Negative	PE(36:4)	$C_{41}H_{74}NO_8P$	2	20.74	<0.05	6.74	1.071

Supplementary Table 3. *Cont.* Summary of non-polar biomarkers with tentative identities that significantly differ between 0 day old and 7 day old wasp extracts. PE = phosphoethanolamine, PC = phosphocholine, TG = triacylglyceride.

M/Z	Average RT (min)	Adduct	ESI Phase	Common Name	Formula	d.f.	*H* value	P value	Fold change	Mass error (ppm)
740.521	4.32	M+H	Positive	PE(36:4)	$C_{41}H_{74}NO_8P$	2	34.98	<0.01	6.59	0.171
740.525	4.68	M-H	Negative	PE(36:3)	$C_{41}H_{76}NO_8P$	2	32.80	<0.01	5.58	1.421
742.526	4.38	M-H	Negative	PE(36:2)	$C_{41}H_{78}NO_7P$	2	30.53	<0.01	4.92	3.3
744.555	5.15	M-H	Negative	PE(36:1)	$C_{41}H_{80}NO_7P$	2	29.04	<0.01	5.58	0.121
762.503	4.32	M+Na	Positive	PE(36:4)	$C_{41}H_{74}NO_8P$	2	35.60	<0.01	5.12	0.985
762.509	4.31	M-Hac-H	Negative	PC(30:1)	$C_{38}H_{74}NO_8P$	2	31.37	<0.01	6.85	3.479
766.535	4.88	M+Na	Positive	PE(36:2)	$C_{41}H_{78}NO_7P$	2	29.73	<0.01	5.50	0.723
782.568	4.32	M+H	Positive	PC(34:1)	$C_{42}H_{82}NO_8P$	2	32.55	<0.01	6.44	0.977
792.707	7.83	M+NH4	Positive	TG(42:2(12:0/18.1/16.1)	$C_{45}H_{82}O_6$	2	41.44	<0.01	-5.73	0.564
794.722	8.76	M+H, M+NA	Positive	TG(46:1(14:0/16.0/16.1)	$C_{49}H_{94}O_5$	2	39.19	<0.01	-5.13	1.214
800.494	5.01	M-HCOO	Negative	PC(35:5)	$C_{43}H_{76}NO_8P$	2	29.52	<0.01	5.45	2
806.566	4.66	M+Na	Positive	PC(36:3)	$C_{44}H_{82}NO_8P$	2	28.97	<0.01	5.29	1.023
816.480	4.85	M-Hac-H	Negative	PC(34:2)	$C_{42}H_{80}NO_8P$	2	32.36	<0.01	6.31	1.994
818.496	4.13	M-Hac-H	Negative	PC(34:1)	$C_{42}H_{82}NO_8P$	2	33.07	<0.01	6.34	1.756
818.721	7.68	M+NH4	Positive	TG(48:3(14:1/16.1/18.1)	$C_{51}H_{92}O_6$	2	22.20	<0.01	-5.51	2.214
820.738	8.90	M+H, M+NA	Positive	TG(48:2(16:0/16.1/16.1)	$C_{51}H_{94}O_6$	2	40.41	<0.01	-5.63	0.864
836.547	3.86	M-Hac-H	Negative	PC(36:6)	$C_{44}H_{76}NO_8P$	2	33.60	<0.01	5.52	2.294
838.561	4.09	M-Hac-H	Negative	PC(36:5)	$C_{44}H_{78}NO_7P$	2	32.19	<0.01	6.35	0.644
840.576	4.38	M-Hac-H	Negative	PC(36:4)	$C_{44}H_{80}NO_7P$	2	33.16	<0.01	6.74	0.006
842.592	4.73	M-Hac-H	Negative	PC(36:3)	$C_{44}H_{82}NO_8P$	2	28.04	<0.01	5.58	0.344
844.608	5.12	M-Hac-H	Negative	PC(36:2)	$C_{44}H_{84}NO_7P$	2	23.88	<0.01	4.92	0.694
844.738	7.90	M+NH4	Positive	TG(50:4(16:0/16.1/18.3)	$C_{53}H_{96}O_5$	2	38.61	<0.01	-6.56	0.864
846.624	5.99	M-Hac-H	Negative	PC(36:1)	$C_{44}H_{86}NO_8P$	2	28.39	<0.01	5.59	1.044
846.753	8.71	M+H, M+NA	Positive	TG(50:3(16:0/16.1/18.2)	$C_{54}H_{98}O_6$	2	42.76	<0.01	-5.99	1.514

Supplementary Table 3. *Cont.* Summary of non-polar biomarkers with tentative identities that significantly differ between 0 day old and 7 day old wasp extracts. TG = triacylglyceride.

M/Z	**Average RT (min)**	**Adduct**	**ESI Phase**	**Common Name**	**Formula**	**d.f.**	***H* value**	**P value (B)**	**Fold change**	**Mass error (ppm)**
849.694	7.90	M+NH4	Positive	TG(50:2(16:0/16.1/18.1)	$C_{53}H_{98}O_6$	2	37.80	<0.01	-4.47	0.259
868.738	7.22	M+NH4	Positive	TG(52:6(16:1/18.2/18.3)	$C_{55}H_{94}O_6$	2	27.62	<0.01	-18.77	0.864
870.753	7.90	M+NH4	Positive	TG(52:5(16:0/18.2/18.3)	$C_{55}H_{98}O_5$	2	35.56	<0.01	-6.05	0.894
872.769	8.74	M+H, M+NA	Positive	TG(52:4(16:0/18.2/18.2)	$C_{55}H_{98}O_6$	2	39.42	<0.01	-6.13	1.164
875.709	7.93	M+NH4	Positive	TG(52:3(16:0/18.1/18.2)	$C_{55}H_{100}O_6$	2	36.55	<0.01	-4.76	0.909
876.799	8.67	M+NH4	Positive	TG(52:2(16:0/18.1/18.1)	$C_{55}H_{102}O_6$	2	31.34	<0.01	-7.43	2.464
894.754	7.15	M+NH4	Positive	TG(54:7(18:2/18.2/18.3)	$C_{57}H_{98}O_5$	2	30.19	<0.01	-5.19	0.514
896.775	8.09	M+NH4	Positive	TG(54:6(18:1/18.2/18.3)	$C_{57}H_{100}O_5$	2	29.67	<0.01	-3.33	4.836
898.784	8.63	M+H, M+NA	Positive	TG(54:5(18:1/18.2/18.2)	$C_{58}H_{104}O_5$	2	27.09	<0.01	-4.52	0.594
901.725	7.78	M+NH4	Positive	TG(54:4(18:1/18.1/18.2)	$C_{57}H_{102}O_6$	2	24.17	<0.01	-3.98	0.559
